# Protocol to perform polysome profiling in primary differentiating murine adipocytes

**DOI:** 10.1016/j.xpro.2025.103799

**Published:** 2025-04-30

**Authors:** Mirian Krystel De Siqueira, Zaynab Nouhi, Yutian Zhao, Siqi Wang, Xinshu Xiao, Xia Yang, Laura Hulea, Claudio J. Villanueva

**Affiliations:** 1 Department of Integrative Biology and Physiology, University of California, Los Angeles, Los Angeles, CA 90095, USA; 2 Maisonneuve-Rosemont Hospital Research Centre, Montréal, QC H1T 2M4, Canada; 3 Département de Biochimie et Médecine Moléculaire, Université de Montréal, Montréal, QC H3C 3J7, Canada; 4 Département de Médecine, Université de Montréal, Montréal, QC H3C 3J7, Canada; 5 Molecular Biology Institute, University of California, Los Angeles, Los Angeles, CA 90095, USA; 6 CAS Key Laboratory of Computational Biology, Shanghai Institute of Nutrition and Health, Chinese Academy of Sciences, Shanghai 200031, China; 7 Department of Molecular and Medical Pharmacology, University of California, Los Angeles, Los Angeles, CA 90025, USA

**Keywords:** Cell Biology, RNA-seq, Metabolism, Gene Expression

## Abstract

Here, we present a protocol for polysome profiling in differentiating adipocytes from the mouse stromal vascular fraction. We describe steps for lysate preparation, ultracentrifugation through sucrose gradients, mRNA isolation, RNA sequencing, and motif enrichment. This protocol enables the analysis of actively translating mRNAs and translational gene regulation by isolating polysome-bound mRNA, revealing insights into protein synthesis during adipogenesis or stimuli responses. Applications include studying translational control in adipocytes, metabolic diseases, and obesity, linking translational regulation to cellular and metabolic phenotypes.

For complete details on the use and execution of this protocol, please refer to De Siqueira et al*.*^1^

## Highlights

• Isolation of murine stromal vascular fraction for primary cell culture
• Polysome profiling of primary adipocytes requires media boost to enhance translation
• On-plate primary adipocyte lysis is essential for high RNA yield
• Translation efficiency analysis of differentiating adipocytes

## Abstract (editor highlights)

Publisher’s note: Undertaking any experimental protocol requires adherence to local institutional guidelines for laboratory safety and ethics.

## Graphical abstract

**Figure:**
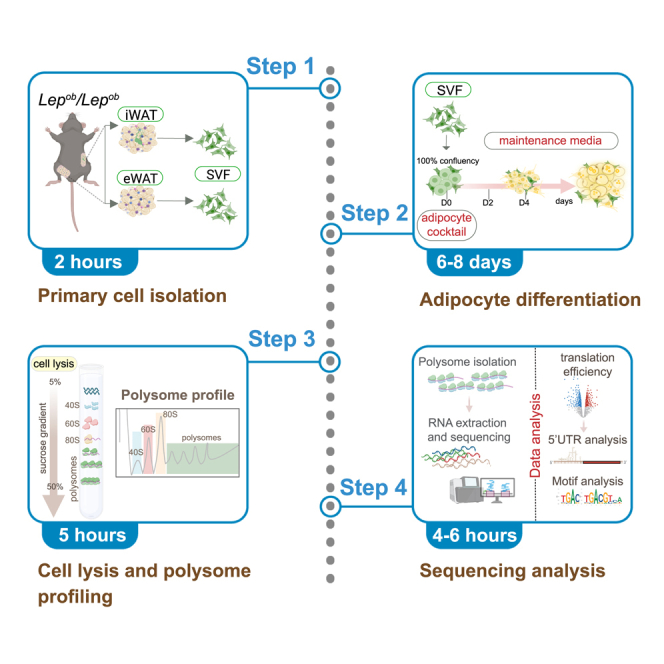

## Before you begin

It would be helpful to work with stable cell lines before optimizing the protocol for primary cells. A cell line with a similar phenotype would be ideal to first test the yield of RNA after lysis in the hypotonic buffer and to assess the quality of the polysome profile. In our case, we performed troubleshooting with 10T1/2 cells before isolating the stromal vascular fraction. In addition to that, if using primary cells from mice an approved animal use protocol will also be necessary.

### Institutional permissions

Adult 12-week-old *Lep*^*ob*^*/Lep*^*ob*^ (stock #000632) male mice can be acquired through Jackson Laboratories. All mice are housed at a maximum of 5 animals per cage in temperature-controlled rooms on a 12-h light/dark cycle and provided water and chow *ad libitum.* All mouse procedures are performed according to animal study proposals approved by the University of California, Los Angeles Animal Research Committee (ARC 2019-066).

## Key resources table

**Table:** 

REAGENT or RESOURCE	SOURCE	IDENTIFIER
**Chemicals, peptides, and recombinant proteins**		

Rosiglitazone	Cayman	Cat# 71740
Insulin	Gibco	Cat #12585014
Dexamethasone	Sigma	Cat# D4902
3-isobutyl-1-methylxanthine (IBMX)	Sigma	Cat# I5879
Type II collagenase	Sigma	Cat# C6885
Gibco DMEM, high glucose, pyruvate	Thermo Fisher Scientific	Cat#11995073
Fetal bovine serum (FBS)	Omega Scientific	Cat#FB-11
Penicillin-Streptomycin (10,000 U/mL)	Thermo Fisher Scientific	Cat#15140122
Invivogen Primocin	Fisher Scientific	Cat#NC9141851
0.5 M EDTA pH 8	Thermo Fisher Scientific	Cat#15575020
Cycloheximide (CHX)	Sigma	Cat#C4859
Bovine serum albumin - fatty acid-free	Gemini	Cat#700-107P
Sodium deoxycholate	Sigma	Cat#D6750
Triton X-100	Sigma	Cat#X1000.5
1 M HEPES pH 7.6	Fisher Scientific	Cat#NC1584172
1 M MgCl_2_	Thermo Fisher Scientific	Cat#AM9530G
1M Tris-HCl pH 7.5	Thermo Fisher Scientific	Cat#15567027
3M Potassium chloride	Thermo Fisher Scientific	Cat#043398.K2
Protease inhibitor	Sigma	Cat#11873580001
RNase inhibitor	Thermo Fisher Scientific	Cat#EO0382
1 M dithiothreitol (DTT)	Thermo Fisher Scientific	Cat#p2325
Sucrose	Fisher Scientific	Cat#BP220-212
Ultrapure distilled water	Thermo Fisher Scientific	Cat#10977015
TRizol LS	Invitrogen	Cat#10296028

**Deposited data**		

Polysome sequencing	Gene Expression Omnibus	GEO: GSE268057

**Experimental models: Cell lines**		

C3H/10T1/2	ATCC	Clone#8, CL-226

**Experimental models: Organisms/strains**		

Mouse *Lep*^*ob*^*/Lep*^*ob*^ (male, 12 weeks old)	The Jackson Laboratory	JAX:000632

**Software and algorithms**		

TRIAX	BioComp	N/A
R and RStudio	R Consortium	https://posit.co/products/open-source/rstudio/
FastQC	Andrews^2^	http://www.bioinformatics.babraham.ac.uk/projects/fastqc/
Salmon	Patro et al.^3^	https://combine-lab.github.io/salmon/
DESeq2	Love et al.^4^	https://bioconductor.org/packages/release/bioc/html/DESeq2.html
RSAT	Santana-Garcia et al.^5^	https://rsat.france-bioinformatique.fr/teaching/RSAT_portal.html

## Materials and equipment

**Table:** Digestion media

Reagent	Final concentration	Volume (mL/mg)
DMEM	N/A	50 mL
Type II collagenase	1 mg/mL	50 mg
Bovine Serum Albumin - fatty acid-free	1%	500 mg
Total	N/A	50 mL

**Table:** Adipogenic cocktail

Reagent	Final concentration	Volume (μL)
Insulin (4 mg/mL)	5 μg/mL	250 μL
Rosiglitazone (10 mM)	1 μM	20 μL
3-isobutyl-1-methylxanthine (IBMX) (500 mM)	0.5 mM	200 μL
Dexamethasone (10 mM)	1 μM	20 μL
Total volume (add complete DMEM to top to 200 mL)	N/A	200 mL

**Table:** Hypotonic buffer

Reagent	Final concentration	Volume (μL/tablet)
Tris-HCl pH 7.5 (1 M)	5 mM	25 μL
KCl (3 M)	1.5 mM	2.5 μL
MgCl_2_ (1 M)	2.5 mM	12.5 μL
Protease Inhibitor cocktail	1x	1/2 tablet
Cycloheximide (100 mg/mL)	100 μg/mL	5 μL
Dithiothreitol (DTT) (1 M)	1 mM	5 μL
RNase Inhibitor	200 U/mL	25 μL
Total volume (add ultra-pure water to top to 5 mL)	N/A	5 mL

Hypotonic buffer without inhibitors (protease inhibitor cocktail, RNase inhibitor, cycloheximide and DTT) can be stored at 4°C for more than 6 months.

Inhibitors should be added fresh on the day of the experiment, before lysing, and kept on ice at 4°C.

**Table:** 10x gradient buffer

Reagent	Final concentration	Volume (mL/tablet)
HEPES pH 7.6 (1 M)	200 mM	2 mL
KCl (3 M)	1M	3.3 mL
MgCl2 (1 M)	50 mM	0.5 mL
Dithiothreitol (DTT) (1M)	1 mM	0.010 mL
Protease Inhibitor cocktail	1x	1 tablet
Cycloheximide (100 mg/mL)	100 μg/mL	0.010 mL
RNase Inhibitor	100 U/mL	0.025 mL
Total volume (add ultra-pure water to top to 10 mL)	N/A	10 mL

10x gradient buffer should be prepared fresh and used right away.

**Table:** Detergent lysis: 10% Triton

Reagent	Final concentration	Volume (mL)
Triton-X-100	10%	1 mL
Ultra pure water	N/A	9 mL
Total volume	N/A	10 mL

10% detergent lysis should be prepared in advance and stored at 20°C–25°C, for up to 2 weeks.

**Table:** Detergent lysis: 10% sodium deoxycholate

Reagent	Final concentration	Volume (mL/g)
Sodium Deoxycholate	10%	1 g
Total volume (add ultra-pure water to top to 10 mL)	N/A	10 mL

10% detergent lysis should be prepared in advance and stored at 20°C–25°C, for up to 2 weeks.

## Step-by-step method details

### Stromal vascular fraction isolation

**Timing: 2 h**

Before the polysome profile can be performed, primary cells must be isolated *ex vivo* and differentiated.

**Figure 1:**
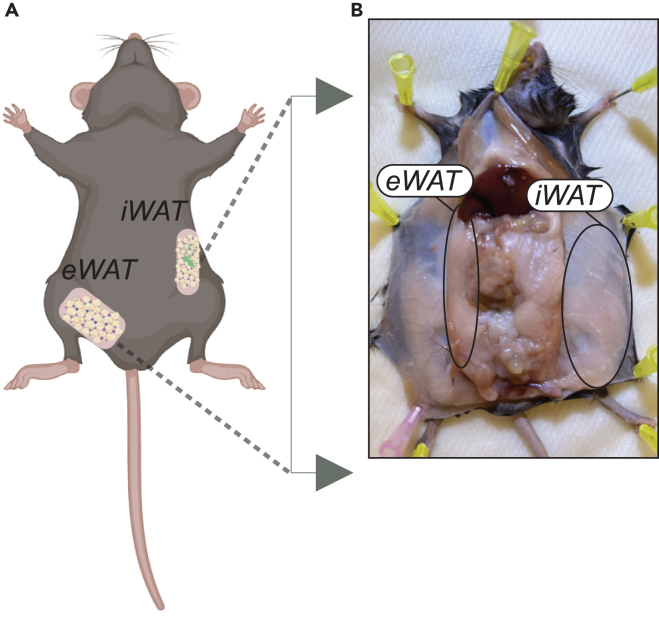
Isolation of adipose tissue depots (A) Mice cartoon illustrating the location of epididymal adipose tissue (eWAT) and inguinal adipose tissue (iWAT). (B) Mice anatomical location of adipose depots. Please note the circles highlighting the epididymal adipose tissue (eWAT) and inguinal adipose tissue (iWAT).

1. Using a sterile scissor/forceps carefully remove fat pads - epididymal adipose tissue (eWAT) and inguinal adipose tissue (iWAT) - from mice and keep the entire fat pad in a cold PBS with 1% Penicillin/ Streptomycin (Pen/Strep) (Figure 1).

**CRITICAL:** Identify and remove lymph nodes from the iWAT using sterile forceps.

2. Mince the adipose tissue pad (2–3 g *Lep*^*ob*^*/Lep*^*ob*^ mice) in a sterile 6-well plate with 1 mL of digestion media (DMEM, 1% BSA, 1 mg/mL type II collagenase) using sharp scissors for 2–3 min.
3. Add 3 mL of digestion media.
4. Incubate samples for 30 min at 37°C with agitation (120 rpm) - every 15 min, pipette the samples up/down to help disrupt the tissue.
5. After digestion, add 1 mL of 2 mM EDTA DMEM to stop digestion and transfer tissues to a 50 mL canonical tube.
6. Add 30 mL of 1% Pen/Strep DMEM to a new canonical tube and attach a 100 μm cell strainer on top. Pre-wash the cell strainer with 1 mL of 1% Pen/Strep DMEM, and filter cell suspension in circular movements to avoid clogging.
7. Centrifuge samples for 10 min, 700xg at 4°C.
8. Carefully aspirate the fat layer and the media. Resuspend the pellet first in 1 mL of 1% Pen/Strep DMEM and then add 9 mL of the media. Pass the cells through a pre-washed 40 μm cell strainer as previously described on step 6.
9. Centrifuge samples for 3 min, 700 xg at 4°C.
10. Resuspend the pellet in 1 mL of complete DMEM media (10% FBS, 1% Pen/Strep, and 50 μg/mL of Primocin).
11. Plate cells in a 15-cm dish with 25 mL of complete DMEM media.

***Note:*** Protocol for *Lep*^*ob*^*/Lep*^*ob*^ mice yielding ∼10^6^ cells per fat pad.

12. Next day wash cells twice with warm 1% Pen/Strep PBS to remove red blood cells and cell debris.
13. Add fresh complete DMEM media.
14. Culture cells until 80% confluency by changing media (complete DMEM) every 2 days.

**CRITICAL:** Cell density should be adjusted at this point between samples. For primary cells, we recommend only one passage to keep adipogenic potential.

### Adipocyte differentiation

**Timing: 6–8 days**

At this step, cells are ready to be differentiated in adipocytes.

15. Day −2: Once cells reach 80–90% confluency replace the complete DMEM media with the induction media (complete DMEM with 5 μg/mL insulin) until confluency (∼2 days).
16. Day 0: Cells should be 100% confluent. Add the complete adipogenic cocktail: 0.5 mM 3-isobutyl-1-methylxanthine, 1 μM dexamethasone, 5 μg/mL insulin, 1 μM rosiglitazone.

***Alternatives****:* Rosiglitazone can be replaced by 20 nM of GW1929.

17. Day 2: Add the maintenance media (5 μg/mL insulin, and 1 μM rosiglitazone in complete DMEM media).
18. Day 4–8: Replace maintenance media every 2 days.

***Note:*** For our experiments, we differentiated cells until day 4 to match the *in vivo* treatment. Cells can be differentiated for longer periods if needed.

### Sucrose gradient

**Timing: 3 h**

After cells have been differentiated/treated, this step outlines the appropriate cell lysis to maintain intact polysomes.

**CRITICAL:** 10% Sodium deoxycholate and 10% Triton-X-100 can be made in advance and stored for 14 days at 20°C–22°C.

**CRITICAL:** Steps 26 to 58 should be performed on the same day.

**CRITICAL:** Place the ultracentrifuge rack, SW41Ti ROTOR at 4°C, and turn on the ultracentrifuge to reach 4°C before starting.

19. Prepare 60% (W/V) sucrose solution in RNase/DNase-free water. Vacuum filter solution with 0.22 μm filter under biological safety cabinet and store in the fridge.
20. Prepare 5% and 50% sucrose gradient using 60% sucrose.
  a. 10x buffer: 200 mM HEPES pH 7.6, 1 M KCl, 50 mM MgCl2, 100 μg/mL cycloheximide, 100 U/mL RNase Inhibitor, 1X Protease Inhibitor, 1 mM DTT in RNase free water.
  b. 5% sucrose gradient (e.g., 50 mL): 4.2 mL of 60% sucrose (w/v), 5.0 mL of 10x buffer, 40.8 mL of ddH_2_O.
  c. 50% sucrose gradient (e.g., 50 mL): 41.7 mL of 60% sucrose (w/v), 5.0 mL of 10x buffer, 3.3 mL of ddH_2_O.
  d. Vacuum filter both 5% and 50% sucrose solutions.
21. To make the gradient use the BioComp short gradient maker protocol for SW41Ti, optimized for short caps.
22. Label 13.2 mL Ultra Clear tubes. Use the marker block SW41 tubes for short caps.
23. Start by loading the 5% sucrose gradient until the indicated mark.
24. Then add the 50% sucrose gradient from below the 5% sucrose gradient (Methods videos S1 and S2).

**CRITICAL:** To have good polysome profiling, it is crucial not to create any bubbles.

**CRITICAL:** To have consistent and accurate 5–50% gradient, it is important not to generate any bubbles or mixing of the 50% into 5% sucrose solution.

25. Keep the gradients in the fridge (4°C) until the samples are ready. Gradients can be prepared in advance and stored at −80°C for a longer time.

**Pause Point:** Gradients can be prepared in advance and stored at −80°C for several months. To thaw, leave them at 4°C the day before the experiment.

Methods video S1. Sucrose gradient: A video demonstration of the preparation of a 5%–50% sucrose gradient, related to step 24

Methods video S2. Capping of sucrose gradient tubes: A video demonstration of the proper cap insertion technique to prevent bubble formation, related to step 24

### Isolation of polysomes

**Timing: 2 h**

In this step cells will be treated and lysed for polysome isolation.

26. Before lysing cells to perform polysome profiling, replace the cell maintenance media from step 18, for 1 h.

***Note:*** Refreshing the media will give a boost in translation and will generate better-defined polysome peaks.

27. Treat the cells with 100 μg/mL of cycloheximide for 5 min, at 37°C.
28. Wash cells twice with ice-cold PBS containing 100 μg/mL cycloheximide.

**CRITICAL:** Cycloheximide inhibits translation elongation by binding to the ribosomal E-site. This step is crucial to prevent ribosomes from dissociating from mRNAs during sample lysis.

29. Resuspend samples in 540 μL of hypotonic buffer: 5 mM Tris-HCl pH 7.5, 1.5 mM KCl, 2.5 mM MgCl2, 100 μg/mL cycloheximide, 1 mM DTT, 1x protease inhibitor, 200 U/mL RNase inhibitor in RNase free water.

**CRITICAL:** Add hypotonic buffer directly to the plate and use a cell scraper to detach the cells. Angle plates at 45 degrees on ice, pipette cells up and down two times to resuspend the cells. Transfer the resuspension into a cold 1.5 mL Eppendorf tube. Estimate the collected volume (EV).

30. Vortex the tubes for 5 s.
31. For each sample, add (i) a volume equal to 5% of EV of 10% Triton-X-100 and (ii) a volume equal to 5% of EV of 10% Sodium deoxycholate.
32. Vortex for 5 s.
33. Centrifuge samples at 16,000 xg for 7 min at 4°C.
34. Transfer the supernatant to a new pre-chilled 1.5 mL tube.
35. Measure OD260 of each sample.

**CRITICAL:** Keep 10% of the sample (50 μL) for input. Add 450 μL ddH2O and 500 μL TRizol LS and store at −80°C.

**CRITICAL:** For the best profile, an OD of 10 to 20 is required.

36. Remove 500 μL from the top of each sucrose gradient and slowly layer 450 μL of cell lysate on top (Methods video S3).

**CRITICAL:** Add the samples very slowly to prevent disrupting the gradient.

37. Weigh and balance the tubes using the hypotonic lysis buffer.
38. Centrifuge at approximately 160,000 xg (36,000 RPM) at 4°C for 2 h using the SW41Ti rotor.

**CRITICAL:** It is essential to have no brake, 0 deceleration, and acceleration.

Methods video S3. Loading of samples into the sucrose gradient: A video demonstration of the precise removal of excess gradient solution and the accurate loading of protein lysate, related to step 36

### Fractionation and polysome profile

**Timing: 1 h**

**Figure 2:**
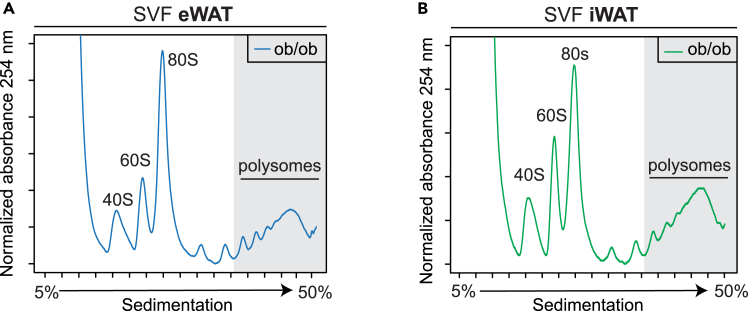
Polysome profile of differentiating adipocytes from eWAT and iWAT (A) Polysome profile of stromal vascular fraction of epididymal adipose tissue (eWAT) at day 4 of adipocyte differentiation. (B) Polysome profile of stromal vascular fraction of inguinal adipose tissue (iWAT) at day 4 of adipocyte differentiation.

After ultracentrifuging the samples in the sucrose gradient, the fractionation step is essential to provide the polysome profile (Figure 2) and also to individually collect each fraction.

39. Carefully place ultracentrifuge tubes on ice.
40. Turn on the UV detector, fraction collector, and computer, and run the fractionator software.

**CRITICAL:** Turn on the fractionator for at least 15 min before starting the fractionation.

***Note:*** Steps 41 to 57 are specific to the TRIAX instrument from BioComp. If using another instrument, follow the manufacturer's instructions.

41. Turn the Frac/Station ON and press the SCAN key.
42. PC ON, boot up TRIAX software.
43. Gibson ON, in the gutter, and programmed for your rack.
44. Review the USER, LED POWER, and SCAN SETUP pages, and go to the graph.
45. Press RINSE and adjust Air pressure until 1 drop per second.
46. Press RINSE then AIR 4 times to clean the system.
47. Apply the rinse adaptor, inject RNase/DNase-free water, and zero the OD.
48. Lube the silicone piston tip and apply it to the piston.
49. Attach gradient to holder cap, load into the holder, and mount holder under the piston.
50. START SCAN on PC.
51. The collector should move to first sample, when the piston starts moving down, press AIR, and hold ON until 2 s after the piston stops.
52. Watch the run develop on the screen.
53. Blow out the last drops.
54. Save run (.csv and .bmp).
55. Add equal volume TRizol LS to each collected fraction.

**Pause Point:** Fractions in TRizol can be stored at −80°C for several months before RNA extraction.

56. If another identical run will follow, there is no need to re-zero.
57. Press RINSE then AIR 4 times to clean the system between each sample and repeat steps 48 to 54.
58. POST-SCAN: after the last run, do a long rinse, drain water from the reservoir and proceed to dry the pump and system with AIR according to the manufacturer’s instructions. (https://biocompinstruments.com/documents/mMtype/category/fractionation).
59. Polysome profiles from the same run can be aligned together in one graph.

**Figure 3:**
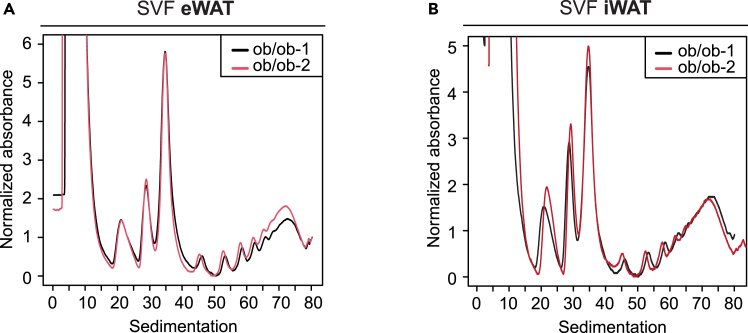
Reproducibility of polysome profiles (A) Biological duplicates of polysome profile of stromal vascular fraction of epididymal white adipose tissue (eWAT) at day 4 of adipocyte differentiation. (B) Biological duplicates of polysome profile of stromal vascular fraction of inguinal white adipose tissue (iWAT) at day 4 of adipocyte differentiation.

***Note:*** Our protocol presents highly reproducible polysome profiles within the same biological group (Figure 3).

### RNA sequencing and differentially translated transcript analysis

**Timing: 4–6 h**

RNA extraction and sequencing were described in De Siqueira et al*.*^1^ Here we define the major steps to analyze the differentially transcribed transcripts using two different pipelines.

***Note:*** To interpret the results, the identification of polysome-specific DEGs is critical to defining highly translating mRNAs in the Rosi-treated samples compared to control samples.

60. Analysis I: Comparisons between total RNA and polysome-associated differentially expressed transcripts.
  a. First conduct quality control of raw sequencing data using FastQC^2^ with default parameters for total RNA and polysome data. Next, map the sequencing reads to the reference mouse genome (mm10) with Salmon.^3^
  b. To define transcripts increased by Rosiglitazone, perform differential gene expression analysis on total RNA and polysome data, respectively, using DESeq2.^4^ Identify genes as significant differentially expressed genes (DEGs) if they pass adjusted *p-*value < 0.05 and |log2FC| > 0.2.
  c. Compare the DEGs of the two datasets using Fisher’s Exact Test. Define overlapping DEGs as genes with tight transcriptional and translational coupling and efficient translation.
  d. Interpret polysome-specific DEGs as candidates for rapid or Rosiglitazone-responsive protein synthesis, independent of transcriptional changes. Consider total RNA-specific DEGs as indicative of translational buffering, where transcripts may be regulated for temporary storage or sequestration against active translation.
  e. Validate the results with proteomic analysis by testing the significant overlap between differentially regulated proteins and transcripts with high translational activity in response to Rosiglitazone treatment.
  f. Use immunoblotting to further validate the proteins that show high translational activity and differential regulation in response to Rosiglitazone in two different adipose depots, respectively.
  g. For complete details on the use and execution of this pipeline, please refer to De Siqueira et al.^1^
61. Analysis II: Identifications of differentially translated transcripts and discovery of enriched motifs.
  a. First perform quality control, alignment, and quantification of the sequencing reads using the same pipeline as “step a” in Analysis I.
  b. To normalize the level of total RNA abundance in polysome fractions, include an interaction term between RNA fractions (i.e., polysome vs. total RNA) and treatment (i.e., Rosiglitazone vs. vehicle) in DESeq2^4^ model.
  c. Next, define the differentially translated transcripts (DTETs) using the criteria of adjusted p-value < 0.05 and |log2(FC)| > 1.
  d. To further explore the sequence patterns, first extract mRNA sequences including 5′-untranslated, coding, and 3′-untranslated regions based on the transcriptome annotations from GENCODE VM33. Then, extract the sub-sequences from DTETs regions by overlapping hexamers using the sliding-window length as 6 nt and step size as 1 nt.
  e. Count the occurrences and frequencies of each hexame, and calculate the enrichment score using Z-test. To define the enriched hexamers between up- vs. down-translated (as background) transcripts, use the enrichment z-score higher than 3 as the cutoff.
  f. Conduct the motif analysis of the enriched hexamers using RSAT^5^^,^^6^ and generate sequence motifs using R package ‘ggseqlogo’.
  g. For detailed description of this pipeline, please refer to De Siqueira et al.^1^

## Expected outcomes

eWAT-iWAT primary cells typically yield an OD of 9-11 from one 15-cm dish. 10T1/2 cells usually present a higher yield (OD 14–25), especially during the late stages of differentiation. If the cell lysis is efficient, with the expected yield, polysome profiling should present very well-defined small (40S), and large subunits (60S), monosomes (80S), and polysomes. Polysome sequencing will highlight the highly translated transcripts that should be reflected at the protein level (proteomics or western blot).

## Limitations

Polysome profiling is a powerful technique for studying translation, but it has several limitations. One major drawback is its low resolution of translation events, as it measures ribosome density on mRNA without providing precise ribosome positions on the transcript. In contrast, ribosome profiling (Ribo-seq) offers codon-level accuracy. Additionally, polysome profiling has a limited dynamic range, making it difficult to detect subtle changes in translation efficiency, particularly in low-abundance transcripts. Another limitation is its lack of functional insights—while it identifies mRNAs associated with ribosomes, it does not confirm whether these transcripts produce functional proteins, necessitating complementary methods like proteomics. Furthermore, polysome profiling is restricted to cytoplasmic translation and does not effectively assess translation occurring in organelles such as mitochondria.

## Troubleshooting

### Problem 1

#### Absence of defined polysome peaks

Since the cells used for this protocol are fully confluent and differentiated, the translation machinery may not be as active as with dividing cells.

### Potential solution

**Figure 4:**
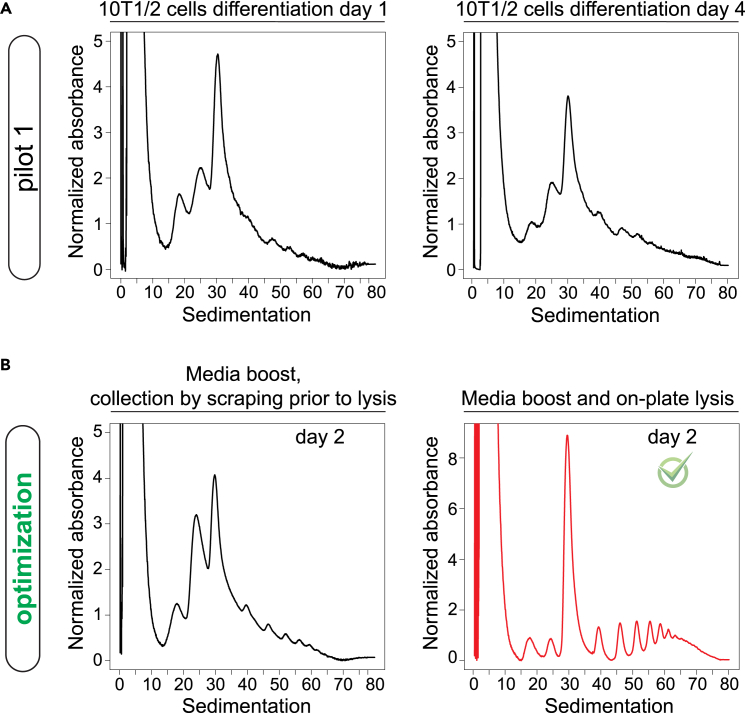
Polysome profile optimization (A) Pilot 1 using 10T1/2 cells. Cells were differentiated for 1 or 4 days. Lysis was done in a tube post-collection. The left image shows the polysome profile on day 1 and the right image shows the polysome profile on day 4 with the absence of defined polysome peaks. (B) Optimization of polysome profile: 10T1/2 cells were differentiated for 2 days and media was replaced 1 h before lysis (media boost). The left image shows the polysome profile of cell lysis in a tube post-collection. The right image shows the polysome profile of cell lysis directly on the tissue culture plate with well-defined polysome peaks.

To boost translation, we recommend adding fresh media 1 h before cell lysis and to perform cell lysis directly in the culture plate (Figure 4).

***Note:*** This step is essential to provide the polysome profiles (Figure 2).

### Problem 2

#### Low OD260 yield after lysis

During lysis of the samples, the dissociation may not be complete.

### Potential solution

To avoid poor lysis, we recommend lysing in plates, and in case of low OD260, we recommend increasing cell quantity. In addition to that, make sure to prepare all reagents fresh, process samples on ice, and make sure to load the samples on the sucrose gradient as soon as possible to avoid RNA degradation.

**CRITICAL:** For the best profile, an OD of 10 to 20 is required.

### Problem 3

#### RNA degradation

Working with RNA might be challenging in terms of RNA degradation. Degradation of RNA can occur in the lysed cell input, prior to loading on the gradient. Therefore, it is essential to verify input RNA quality if polysome RNA quality is low. RNA extraction and sequencing were performed as described in De Siqueira et al.^1^

### Potential solution

To avoid RNA degradation, we recommend using RNase/DNase-free water for all solutions, filtering all solutions, and thoroughly cleaning the workspace with 2% SDS and 70% EtOH.

## Resource availability

### Lead contact

Further information and requests for resources or reagents should be directed to the lead contact, Claudio Villanueva (cvillanueva@ucla.edu).

### Technical contact

Technical questions on executing this protocol should be directed to and will be answered by the technical contact, Laura Hulea (laura.hulea@umontreal.ca).

### Materials availability

This study did not generate new unique reagents.

### Data and code availability

All original code has been deposited at Zenodo at https://doi.org/10.5281/zenodo.13926724 and is publicly available.

## Acknowledgments

This work was supported by the NIH (UL1TR001881 and R01DK103930 to C.J.V.), the American Heart Association (23PRE1018867 to M.K.D.S.), the 10.13039/501100000024Canadian Institutes of Health Research (PJT 165901 to L.H.), the Canadian Funds for Innovation (JRELF 39246 to L.H.), and the Fonds de Recherche du Québec-Santé Junior 2 Investigator Award (to L.H.).

## Author contributions

M.K.D.S. established the adipose stromal vascular fraction polysome profiling. Z.N. optimized and established the polysome profiling for the 10T1/2 cells. X.Y. supervised, advised, and discussed data generated by Y.Z. X.X. supervised, advised, and discussed data generated by S.W. L.H. supervised, advised, and discussed the polysome profiling data. M.K.D.S., Z.N., L.H., and C.J.V. drafted the manuscript. M.K.D.S., Z.N., L.H., and C.J.V. revised the manuscript.

## Declaration of interests

The authors declare no competing interests.
